# The impact of total body water on breath alcohol calculations

**DOI:** 10.1007/s00508-020-01663-4

**Published:** 2020-05-25

**Authors:** Gregor S. Reiter, Markus Boeckle, Christian Reiter, Monika H. Seltenhammer

**Affiliations:** 1 Center for Forensic Medicine, Medical University of Vienna, Sensengasse 2, 1090 Vienna, Austria; 2 Department of Ophthalmology and Optometry, Medical University of Vienna, Vienna, Austria; 3 Karl Landsteiner University of Health Sciences, Dr. Karl-Dorrek-Straße 30, 3500 Krems, Austria; 4 Department of Psychiatry and Psychotherapy, University Hospital Tulln, Alter Ziegelweg 10, 3430 Tulln, Austria; 5 Division of Life Stock Sciences, University of Natural Resources and Life Sciences, Vienna, Austria

**Keywords:** Bioelectrical impedance assessment (BIA), Blood alcohol concentration (BAC), Forensic back calculation, Body surface area (BSA), Gender-specific differences

## Abstract

Due to a legislative amendment in Austria to determine breath alcohol (BrAC) instead of blood alcohol (BAC) in connection with traffic offences, many results of blood alcohol calculations were simply converted using distinct conversion factors. In Austria, the transformation of BAC to BrAC was carried out by using a factor of 1:2000, which, however, is commonly known to be too low. Noticing the great demand for a calculation method that is not exclusively based on blood alcohol, a formula for calculating breath alcohol based on blood alcohol was published in 1989, but in which the body surface area (BSA) was considered the most important influencing variable. In order to refine this new method, a liquor intake experiment was conducted combined with measurements of total body water (TBW) as an additional variable, using hand to foot bioelectrical impedance assessment (BIA). The test group comprised 37 men and 40 women to evaluate the accuracy of TBW and BSA as an individual parameter for alcohol concentration. The correlation coefficient of BrAC with TBW was constantly higher than with BSA (maximum = 0.921 at 1 h and 45 min after cessation of alcohol intake). These results are valid for both men and women as well as in a gender independent calculation. Hence, for an accurate back calculation of BrAC adjusted values of eliminations rates had to be found. This study describes mean elimination rates of BrAC for both men (0.065 ± 0.011 mg/L h^−1^) and women (0.074 ± 0.017 mg/L h^−1^). As previously shown women displayed a significantly higher elimination rate than men (*p* = 0.006).

## Introduction

Evidence of alcohol in the breath was already mentioned in the nineteenth century [1, 23]. In 1927 a potential relationship between blood alcohol concentration (BAC) and breath alcohol concentration (BrAC) was described by Bogen [4]. Soon afterwards, Liljestrand and Linde presented a conversion factor of 1:2000 from BAC to BrAC [23, 29]. This specific conversion factor, commonly shortened to Q, has already been discussed and revised in many different publications [10, 16, 21, 26, 33]. Basically, the threshold of blood alcohol level punishable under Austria’s legislation is 0.5 parts per thousand, which corresponds to 0.25 mg/L BrAC. Due to practical reasons, the usage of BrAC has a more important role than BAC in Austria because of the 13th amendment of the Road Traffic Act (StVO) in 1986 in which blood sampling was almost entirely replaced by breath testing [38]. While BrAC is the most frequently used measurement of alcohol intoxication in Austria, the usage and units of BrAC have yet to be standardized, as various units are applied in the literature [7, 13, 34, 35]. In order to simplify the comparison with Austria’s legislative text it was decided to use the unit mg/L for BrAC in this study.

In the course of Widmark’s work and back calculation of BAC, his equation became the gold standard for daily routine of forensic medicine in Austria. Given the fact that the enhancements of the Widmark equation by Watson et al. [41] or Seidl et al. [37] were not generally accepted in Austria, another formula for calculating BrAC was established in 1989 by Fous et al., which is still frequently used [14]. This formula uses body surface area (BSA) as the main influencing variable to calculate BrAC considering the height and weight of the tested person.

The formula for BrAC is as follows:

$$c_{p}=gAlk\div 100\times [1.5645-0.1524\times ES-0.3961\times BSA-0.05m^2]$$

where c_p_ stands for BrAC at time zero measured in g/l, gAlk for the amount of alcohol intake measured in grams, ES for the contents of the stomach, which has a value between 0 for empty stomach and 1 for full stomach and BSA [14].

On closer inspection of the study design of the original publication it is noticeable that the study did not include women [14]. Even though gender-specific differences in terms of metabolism of alcohol are well known, the formula is still used in Austria’s courts. In particular, the potential problem then becomes evident especially if you don’t just look at BSA: while men and women of the same size and weight are equal in BSA per se, the volume of total body water (TBW) significantly differs; however, TBW is the most important feature to be aware of when re-evaluating the formulas considering the sex-specific difference because of the following reason: ethanol is a hydrophilic substance and therefore mainly distributed in the water-containing compartments of the body [7, 17, 18, 22, 25]. In respect to this matter, there is a great demand for establishing a gender-equitable coefficient as a corrective element in order to adjust the varying results in accordance with the different body constitution between men and women. The underlying idea was already applied by Seidl et al. when they tried to update the calculation of BAC performing the method of bioelectrical impedance assessment (BIA) by means of a foot to foot BIA [37]. After several improvements, multifrequency hand to foot BIA can provide reliable data for TBW and improve the accuracy in people with a body mass index (BMI) >34 kg/m^2^ [15]. Based on these considerations an improvement of the calculation of BrAC has to be found which does not only include the height and weight of the tested person, but also an individual volume of distribution for alcohol.

With this in mind the first and original focus of this study was to test the potential improvement of BrAC calculations based on TBW compared to BSA.

Replacing a judicial system based on BAC with BrAC also means that the controversial conversion factor Q would lose its importance, which, however, may consequently imply a different problem. Many studies have already shown elimination rates of BAC per hour [11, 21, 24]. From today’s scientific point of view women show a significantly higher elimination rate than men [11, 24, 34, 40].

Hence, the second focus is to pay attention to determination of hourly elimination rates of BrAC not only for men but also for women.

## Material and methods

### Subjects and conditions

After approval from the local ethics committee (1527/2014), drinking experiments were conducted as described earlier [16]. The test group comprised 77 individuals. In order to avoid any possible bias, we balanced our collective groups of both men and women with a wide range of BMIs and ages. There were no restrictions regarding body constitution. Reasons for excluding participants were an already existing liver disease, any form of epilepsy, being below legal drinking age in Austria, and possible pregnancy. Moreover, participants were also asked to disclose any possible alcohol dependency, which might influence the elimination rate.

The experimental set-up was as follows: all participants were asked to eat a standardized meal of 100 g of pasta (weight when uncooked) with tomato sauce more than 4 h before coming to the experiment as the only meal of that day prior to the experiment. Foods containing oil or fat were not permitted to be added to the meal. Only in this way could an empty stomach and comparable conditions for all participants be guaranteed.

Every participant was administered the same amount of alcohol, 375 mL of white wine (12 vol%), corresponding to 36 g alcohol, which had to be finished with steady intake within 15 min. Based on the Widmark equation, the drink-drive limit is reached in most participants with 36 g of alcohol. We exemplify calculations based on fictive cases for clarification:

Given the Widmark equation:

$$c=A/(p\times r),$$

where c stands for BAC, A for the amount of alcohol intake in grams, p for the weight of the person in kg and r for the correction factor, which is described as the ratio of total body ethanol and blood ethanol concentration, commonly chosen as 0.7 for men and 0.6 for women [20, 37, 39].

We take a 60 kg woman and a 85 kg man as an example:

$$c=36/(60\times 0.6)$$

which results in a concentration of 1 g/kg;

$$c=36/(85\times 0.7)$$

which results in a concentration of 0.605 g/kg.

These assumptions were made taking into account a theoretical bioavailability of ethanol of 100% and an overall distribution at time zero. Although these can be fictitious values, many judgments are based on this area of alcohol consumption. Although these may be fictional values, many argumentative judgments are found in this range of alcohol uptake. All of the participants finished the drink within the given time conditions.

### Breath alcohol concentration analysis

We analyzed BrAC by means of the Dräger Alcotest 7110 MKIII A (Drägerwerk AG & Co. KGaA, Lübeck, Germany), which is the standard model used by the Austrian police. This model uses an infrared optical measurement system as well as an electrochemical system for measuring the alcohol concentration from a given breath sample. Every participant was asked to give breath samples before drinking to ensure zero alcohol concentration and further two samples every 30 min after the start of drinking until the end of the experiment after 150 min. These standardized conditions guaranteed a realistic and reliable measurement, which would also be used accordingly in court. Within the framework of this scientific study we did not use the lower BrAC value of two consecutively taken samples, which is usually used in court but calculated the mean of the two values instead in order to improve the scientific accuracy as main purpose.

### Bioelectrical impedance assessment

After taking the second breath samples, we determined participants’ TBW applying hand to foot BIA with the Nutriguard‑M (Data Input, Pöking, Germany) while keeping the conditions for reliable analysis by asking subjects to maintain a steady lying position for 10 min before taking the measurement. To ensure equal conditions the BIA was conducted by the same examiner throughout the whole experiment. None of the participants used the toilet before TBW measurement. The TBW was determined with multifrequency BIA with frequencies of 5 kHz, 50 kHz and 100 kHz.

### Calculation of derived variables

The calculation of BSA was performed using the formula described by Fous et al. [14], which is the same as that already presented by Du Bois and Du Bois [12] in 1916:

$$BSA=0.007184\text{m}^2 \times \text{Weight}^{0.425\,\text{kg}} \times \text{Height}^{0.725\,\text{cm}}$$

To remove the state of resorption and to have a better comparison with the formula from Fous et al., BAC at time zero (BrAC_0_) had to be calculated. In doing so, BrAC measurements at 90 min and 150 min were used for each participant and integrated at 0, with a largely zero-order kinetics of alcohol elimination being assumed. A safety time distance after the peak BrAC and the moderate amount of intake before changing to a different kind of kinetics at a low alcohol concentration was taken into account [18, 21, 31, 32].

The individual elimination rate of ethanol was calculated using the same measuring points and assumptions as in the calculation of BrAC_0_. Individualized elimination rates were calculated by subtracting the value of the BrAC at 150 min from the BrAC at 90 min.

The calculation of TBW was executed by means of the software NutriPlus 5.4.1. (Data Input). After the measurement all values of TBW were recorded directly from the software.

### Statistical analysis

Gender-specific differences in breath alcohol elimination rates per hour were tested using a two-sample *t*-test. In order to identify correlations between BrAC and biomedical parameters, Pearson product-moment correlation coefficients or Spearman’s rho dependent of the distribution of the variables were calculated. The normality of the data was checked via a Shapiro-Wilk normality test. Additionally, we calculated multivariate regression models via linear models (LM) in order to identify predictors for BrAC_0_, in particular to clarify whether BSA or TBW is the better predictor for BrAC_0_. The BrAC_0_ was determined by interpolating the slope between 90 min and 150 min. Additional candidate variables used were sex, age, body weight, and body size. Corrected Akaike information criteria (AIC_c_) were used to select the most parsimonious model [5]. Significant differences between models were calculated using ANOVA. All analyses were calculated with IBM SPSS Statistics for Mac v. 21 (IBM Österreich, Vienna, Austria). All residuals of the models conformed to normality. The alpha level was set at 0.05 (two-tailed). Descriptive statistical values are presented in mean ± standard deviation.

## Results

The sample comprised a total of 77 (40 female) healthy white individuals with a mean age of 31.1 ± 11.88 years, mean height of 174.14 ± 8.84 cm, and body weight of 71.87 ± 17.34 kg (Table 1).

**Table 1:** Descriptive statistics of participants

		Mean	Standard deviation	Minimum	Maximum
All (*N* = 77)	Age (years)	31.09	11.88	18	60
	Body size (cm)	174.14	8.84	155	194
	Body weight (kg)	71.87	17.34	47.6	140
Male (*N* = 37)	Age (years)	30.62	11.88	21	60
	Body size (cm)	181.19	5.28	166	194
	Body weight (kg)	80.35	15.67	57	140
Female (*N* = 40)	Age (years)	31.52	12.02	18	55
	Body size (cm)	167.63	5.99	155	178
	Body weight (kg)	64.03	15.08	47.6	127

**Fig. 1:**
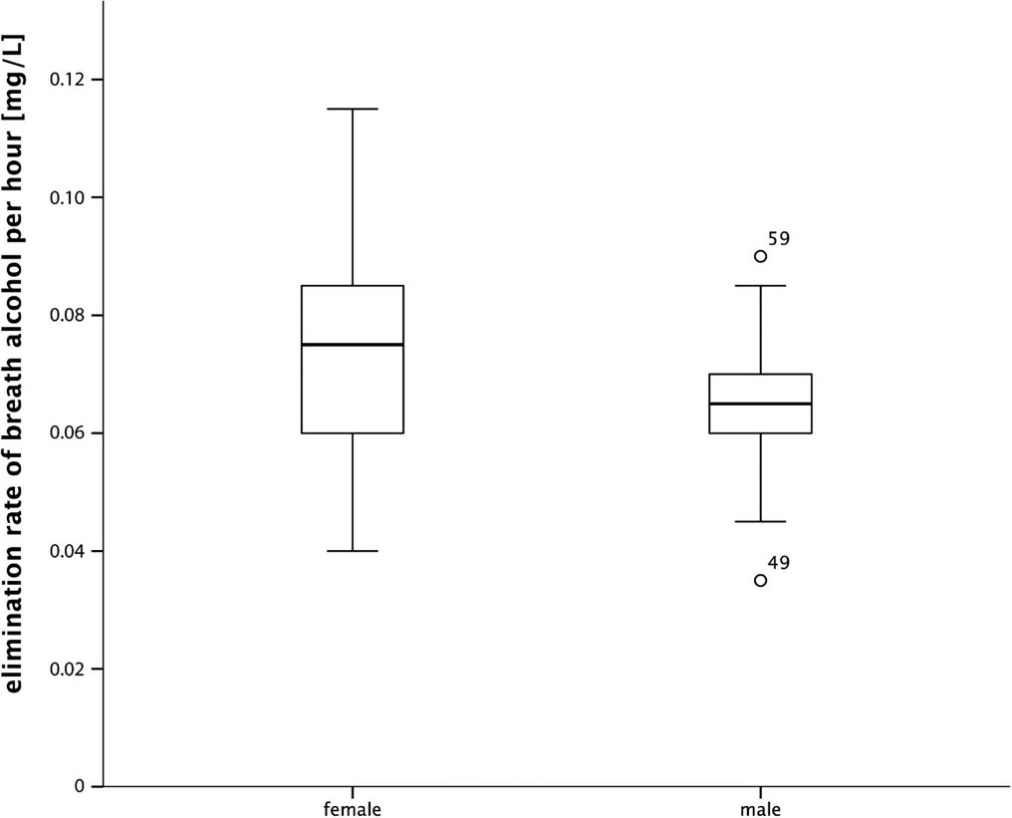
Sex-specific elimination rate of breath alcohol per hour. The median is represented by *bold black lines*, the upper and lower quartiles are the upper and lower border of the *boxes*, the range of data excluding outliers are demarcated by *whiskers*, while statistical simple outliers (cases 49 and 49 are just within one and a half interquartile range) are represented by *circles* in combination with data point identification number

The measured sample had a mean TBW of 37.18 ±7.53 L, a BSA of 1.854 ± 0.225 m^2^, and a BrAC_0_ of 0.358 ± 0.093 mg/L. The mean BrAC_0_ for men was 0.291 ± 0.044 mg/L, whereas the mean BrAC_0_ for women was 0.421 ± 0.084 mg/L. The mean volume of TBW for men was 43.549 ± 4.935 L and 31.28 ± 3.743 L for women. The elimination rate of breath alcohol per hour was 0.0699 ± 0.015 mg/L h^−1^. There was a sex-specific difference in the elimination rate (T_75_ = 2.847; *p* = 0.006; *N* = 77) whereby the male elimination rate was 0.065 ± 0.011 mg/L h^−1^ while the female elimination rate was 0.074 ± 0.017 mg/L h^−1^ (Fig. 1).

Data were not normally distributed for any of the variables, therefore, the results of Spearman’s rho are presented. The BrAC_0_ correlated significantly in the complete sample with TBW (r_s_ = −0.889; *p* ≤ 0.001; *N* = 77) and BSA (r_s_ = −0.865; *p* ≤ 0.001; *N* = 77). When correlations were calculated in the female sample BrAC_0_ showed a negative correlation with TBW (r = −0.835; *p* ≤ 0.001; *N* = 40) and BSA (r = −0.801; *p* ≤ 0.001; *N* = 40). We found similar correlations in males (BrAC_TBW: r = −0.759; *p* ≤ 0.001; *N* = 37; BrAC_BSA: r = −0.682; *p* ≤ 0.001; *N* = 37). The correlations were not significantly different (*p* > 0.05) between sexes.

We found significant correlations between TBW as well as BSA with breath alcohol concentration at time zero min, 90 min, 120 min, and 150 min in the complete sample but also for the female and male subsets (see Table 2).

**Table 2:** Spearman_Rho correlations between breath alcohol (BrAC) and total body water (TBW) as well as BSA, all highly (*p* < 0.001) significant

			BrAC_0_	90min	120min	150min
All (*N* = 77)	TBW	r_s_	−0.889	−0.919	−0.921	−0.901
	BSA	r_s_	−0.865	−0.897	−0.899	−0.879
Female (*N* = 40)	TBW	r	−0.827	−0.896	−0.909	−0.884
	BSA	r	−0.804	−0.852	−0.868	−0.829
Male (*N* = 37)	TBW	r	−0.682	−0.763	−0.733	−0.693
	BSA	r	−0.652	−0.745	−0.714	−0.684

The final model in the model selection process for predicting BrAC_0_ based on TBW was the model including TBW alone with an AIC_c_ of −470.75. The BrAC_0_ is best predicted by TBW (F_1.75_ = 234.257; *p* ≤ 0.001), with a large influence of TBW on BrAC_0_ (β = −0.011; t = −15.305; *p* ≤ 0.001). The final model for predicting BrAC_0_ based on BSA results in a significant influence of BSA (F_1.74_ = 84.103; *p* ≤ 0.001) and sex (F_1.74_ = 11.704; *p* ≤ 0.001) with an AIC_c_ of −468.55. The BSA has a large influence (β = −0.285; t = −9.171; *p* ≤ 0.001) and females showed a higher elimination rate than males (β = −0.047; t = −3.421; *p* ≤ 0.001). When calculating a model with the influence of BSA (AIC_c_ = −459.41) on BrAC_0,_ we found just such an influence (F_1.75_ = 191.913; *p* ≤ 0.001) with a large effect (β = −0.353; t = −13.853; *p* ≤ 0.001). When comparing the three models TBW best explains the variance of BrAC_0_ data (*p* ≤ 0.05).

## Discussion

In this experiment we divided the results into two parts. First, we described the superiority in the correlation between BrAC and TBW over the correlation of BrAC and BSA, and second, we investigated mean gender-specific elimination rates of BrAC and confirmed that women have a higher elimination rate than men. Furthermore, there is also strong evidence that women are more likely to have less alcohol dehydrogenase (ADH) activity in the stomach and, consequently, lower first-pass metabolism. As a consequence, peak alcohol concentration is higher in women [2]. This pharmacokinetic difference is mentioned as a possible reason for a particular vulnerability of women to alcohol [2, 30]. Together with a lower level of ADH activity, females have a smaller volume of distribution [2, 20]. These differences in alcohol pharmacokinetics were already involved in the Widmark equation with the correction factor *r*. When measuring BAC, different methods tried to update this correction factor [36, 37, 41].

Accurate results for TBW can be generated using hand to foot BIA [15]. When comparing BIA with isotope dilution, the results are very similar and the correlation coefficients are higher than those presented by the calculation by York and Hirsch as well as Watson et al. [42, 43]. The underestimation of the Watson et al. calculations has already been previously described [8]. Alternative calculations for TBW have been published and it has been shown that the mean volume of TBW varies in different populations based on ethnicity, gender and other factors [3, 6, 19, 25, 27, 28, 43]. In our study the mean TBW measured with BIA was 43.549 ± 0.811 L for men and 31.28 ± 0.592 L for women. It is stated that the percentage of TBW decreases with age [25], whereas the volume of TBW seems to be relatively stable [6, 9]. Possible intraindividual differences of TBW can be attributed to physical training, menstruation and other factors [37]. For this purpose, measuring TBW for each individual seems to be highly recommended and should be in temporal proximity to BrAC testing to find a highly significant influencing factor of BrAC, a claim which is underlined by the high variation in the volume of TBW.

In Austria, a common way to calculate BrAC is the application of the equation of Fous et al. which uses BSA as the major body influencing factor [14]. According to our results, our strong suggestion is that BSA, which is calculated using height and weight alone, is not as useful as a directly measured individual-specific factor that also includes body constitution, although Hume and Weyers showed a high correlation between TBW measured using tritium and BSA [19]. Furthermore, the use of TBW in order to update the Widmark equation for calculating BAC was tried in the 1990s and 2000s [37], but neither TBW nor any other formulas have been used to update BrAC.

In all our tests BSA as well as TBW were highly correlated to BrAC. Both in the gender independent and the gender dependent calculation the correlation coefficients between TBW and BrAC were higher than between BSA and BrAC.

When relying on equations for calculating the alcohol concentration, the result will always be calculated for a hypothetical value at time zero, which excludes the degradation of ethanol. Due to the high variability of alcohol resorption, it is widely accepted that it is best to use alcohol calculations after the resorption period is over. Therefore, it is necessary to know the elimination rates of alcohol to estimate the concentration at a different time than that when measured. The theoretically assumed elimination rates of BAC, which are weighted differently in Austrian criminal law depending on the offense, are between 0.10 and 0.20 g/kgh^–1^; depending on the type of offense. Still, it is well known that elimination rates can be higher, especially for women [11, 21]. Different elimination rates for BAC were demonstrated and verified in several experiments [11, 24, 21]. When applying blood alcohol testing these results are useful enough; however, as soon as breath alcohol testing is carried out, new values have to be utilized in order to avoid bias from the conversion process. Unfortunately, the units of BrAC differ in many countries. Pavlic et al. as well as Dettling et al. published breath alcohol elimination rates using the unit mg/L h^−1^, which was also performed in this study [11, 34]. The mean elimination rate for both sexes in our study was 0.0699 ± 0.002 mg/L h^−1^. For men the mean elimination rate was 0.065 ± 0.002 mg/L h^−1^, for women 0.074 ± 0.003 mg/L h^−1^. These results are quite comparable with those already described and can definitely be used for more accurate elimination rates of breath alcohol.

In contrast to an improved accuracy when describing BrAC, for practical reasons the usage of BSA will still dominate over the TBW measurement using BIA. Nevertheless, the improvements of BIA in recent years may lead to a reconsideration of this issue in the future.

## Conclusion

In Austria blood alcohol was previously the main means of alcohol testing, but after changing the law to allow breath alcohol testing, most of the values were converted to breath alcohol by simply applying a conversion factor of 1:2000. This is the general regulation in Austrian law [38]. In any case, for a more scientific and accurate view there is a huge demand for new values for breath alcohol. In 1989 Fous et al. published an equation for breath alcohol using body surface area as the main influencing variable, which is still frequently used in Austria [14]. In this study we showed the superiority of total body water compared to body surface area. Taking into account that Seidl et al. already tried to use total body water for a more accurate update of the Widmark equation [37], to the authors’ best knowledge no one has shown these results for breath alcohol testing or a different formula.

To fully avoid the bias of converting BAC to BrAC new elimination rates for breath alcohol have to be found. We found overall mean elimination rates of 0.0699 ± 0.002 mg/L h^−1^ with the mean elimination rates of 0.065 ± 0.002 mg/L h^−1^ in men and 0.074 ±0.003 mg/L h^−1^ in women. The results from this study are comparable with those in the current literature. The expectation that women have a significant higher elimination rate than men could be confirmed (*p* = 0.006).

We were able to show the applicability of TBW in BrAC calculations. Future research in the field of breath alcohol testing should focus on the enhancement of the accuracy of breath alcohol testing and keeping susceptibility to errors as low as possible in order to create a reliable basis for legislation.
